# Laboratory-measured functional abilities and refereeing quality in elite football referees

**DOI:** 10.3389/fspor.2026.1987062

**Published:** 2026-10-16

**Authors:** Ivan Krakan, Josip Tomasko, Ivan Belcic, Dario Novak

**Affiliations:** 1 Faculty of Kinesiology, University of Zagreb, Zagreb, Croatia; 2 Croatian Football Federation, Zagreb, Croatia; 3 Faculty of Civil Engineering, University of Zagreb, Zagreb, Croatia; 4 Institute of Sport Science and Innovations, Lithuanian Sports University, Kaunas, Lithuania

**Keywords:** aerobic capacity, decision-making, elite referees, maximal oxygen uptake, refereeing quality

## Abstract

**Introduction:**

The aim of this study was to determine whether functional abilities are associated with the quality of refereeing in elite football referees.

**Methods:**

Seventeen referees from the highest Croatian national competition represented the complete referee list for the 2022/2023 season. One referee sustained an injury during sprint testing and did not complete the physiological assessment. The final analytical sample consisted of 16 referees. Laboratory testing was performed in January 2023, between the first and second parts of the competitive season. The analysed variables were maximal treadmill speed, speed at the ventilatory threshold, maximal heart rate, heart rate at the ventilatory threshold, absolute and relative maximal oxygen uptake, and relative oxygen uptake at the ventilatory threshold. Refereeing quality was determined from official supervisor grades across the season, including the subsequent review of those grades by the national Referees Committee.

**Results:**

Mean relative maximal oxygen uptake was 51.71 ± 4.28 mL/kg/min, maximal treadmill speed was 18.50 ± 1.03 km/h, and the mean refereeing quality grade was 8.21 ± 0.26. No statistically significant association was found between the examined functional variables and season-long refereeing quality. The full-sample Spearman analysis showed the same overall pattern.

**Discussion:**

The small analytical sample limits the detection of weak and moderate associations. The study hypothesis was not supported.

## 1 Introduction

Football match activity is intermittent and includes repeated changes between lower- and higher-intensity work (1, 2). Referees must follow changes in match pace while maintaining a position that allows correct decisions. Football refereeing places simultaneous physical and cognitive demands on the official. A football match is controlled by the main referee with the support of two assistant referees and the fourth official. Training and conditioning research in football was traditionally focused on players, while referees received less attention. The referee still has to follow the same match, react to the same changes in pace and obtain a position that allows the correct decision to be made. Football refereeing is demanding because physical and mental work are present at the same time. Recent reviews confirm high physical and physiological demands, with differences related to competition level, referee role and match intensity (3, 4). Main referees in high-level matches commonly cover approximately 9–12 km and perform repeated high-intensity activities and spend substantial periods at high relative heart rates (4). Studies on professional Spanish referees and elite referees monitored across many matches report the same need to repeatedly respond to high running and physiological demands (5, 6).

Referees have to maintain endurance, speed and agility throughout the match and are generally older than the players they follow. Maintaining aerobic fitness is important for referees who want to remain at the highest competition level. Match demands are not identical for all officials. Main and assistant referees show different external and internal loads, which supports a separate approach to their physical preparation (7). Good physical preparation also allows the referee to reach an appropriate position before a decision has to be made. Referees need to remain close enough to play to observe infringements and technical-tactical events without interfering with the game. Positioning has been related to the accuracy of decision-making in top-level football (8). Recent work suggests that greater viewing distance does not always reduce decision accuracy, but it can reduce certainty in the decision, which still gives practical importance to timely movement and positioning (9). The activity pattern of refereeing is intermittent. Repeated short high-intensity runs are combined with periods of lower-intensity recovery, and this structure has direct consequences for training (10). A recent systematic review showed that many physical fitness tests are used in football referees, but relationships between test performance and match performance are not consistent across studies (3). A high test value can be important for meeting match demands without necessarily separating better and poorer refereeing quality among already selected elite referees. Aerobic capacity remains a central part of referee fitness because it supports movement throughout the match and recovery between repeated high-intensity actions (11). Functional abilities allow referees to keep the pace of the game, adjust their position and react quickly in dynamic situations. Physical preparation also reduces the chance that fatigue becomes a limiting factor when attention and rapid decision-making are required.

The relationship between physical fitness and decision quality is not fully consistent in recent research. Castillo-Rodríguez et al. found that physical fitness was related to match running performance and the percentage of successful decisions in football referees (12). Refereeing quality is also affected by experience, decision-making strategy, attention and the response to pressure. Expert and novice referees differ in several aspects of disciplinary decision-making (13), while anxiety and match-like pressure can change the cognitive processes used during refereeing decisions (14). Functional abilities should be treated as an important part of refereeing performance, but not as the only factor that determines the final quality of refereeing.

The aim of this study was to determine the association between laboratory-measured functional abilities and season-long refereeing quality in referees from the highest level of Croatian football. The hypothesis was that better functional ability test results would be significantly associated with better refereeing quality.

## 2 Materials and methods

### 2.1 Participants

The initial sample consisted of 17 referees (age 34.53 ± 5.69 years, height 186.61 ± 5.09 cm, body mass 84.81 ± 5.72 kg) officiating under the Croatian Football Federation in the highest national competition. They represented 100% of the referees eligible to officiate in the highest Croatian league during the 2022/2023 season. One referee sustained an injury during sprint testing and did not complete the physiological assessment. The final analytical sample consisted of 16 referees. The Referees Committee of the Croatian Football Federation determines the referee list each year based on refereeing grades from the previous season. At the beginning of each season, referees must also satisfy elimination criteria based on UEFA and FIFA instructions and the normative times prescribed for individual test categories. A valid medical examination and compliance with the permitted body-mass criteria prescribed by the applicable health regulations for football referees were required before physical testing. Ten referees were listed by UEFA and/or FIFA, while seven officiated only in competitions under the Croatian Football Federation. The referees had 17.31 ± 5.49 years of total refereeing tenure. Median refereeing experience in the highest Croatian league was 6.0 years (IQR 3.5–8.25; range 1–23 years).

### 2.2 Study design and testing procedure

The study was approved by the Ethics Committee of the Faculty of Kinesiology, University of Zagreb (protocol 19/23). All referees who participated in the study signed a written informed consent form before testing.

Testing was divided into laboratory assessment and evaluation of refereeing quality during official competition. Laboratory testing was performed in January 2023, between the first and second parts of the 2022/2023 competitive season. Refereeing quality was calculated from official match grades obtained across the entire competitive season and included matches officiated before and after laboratory testing. Before the second part of the season, all referees included in the analysis had passed the official physical fitness tests required for eligibility to officiate. During the competitive season, the referees followed prescribed training programmes and submitted their weekly training records to the conditioning coordinator. Laboratory testing was conducted at the Diagnostic Centre and the Laboratory for Sports Games of the Faculty of Kinesiology, University of Zagreb, under standardised conditions and according to a predefined testing protocol. Testing dates were arranged in cooperation with the Referees Committee. Match appointments during the same week were considered when scheduling the tests, and every referee was tested at least three days after an officiated match.

### 2.3 Assessment of functional abilities

Functional abilities were assessed using the KF1 progressive spiroergometric treadmill protocol, a standard protocol used at the Sports Diagnostic Centre of the Faculty of Kinesiology, University of Zagreb (15). Testing was performed on a Run Race Competition HC1200 treadmill (Technogym) in a closed and ventilated laboratory at 18–21 °C. All participants had previous experience running on a treadmill, so no separate familiarisation session was required. Heart rate was monitored using Polar RS800 telemetry (Polar Electro, Finland). Ventilatory and metabolic variables were continuously recorded using a Quark b automated breath-by-breath gas-analysis system (Cosmed, Italy). A respiratory mask covered the nose and mouth and was connected to a bidirectional turbine. Before each test, flow was calibrated using a 3-L syringe and the gas analysers were calibrated using a known gas mixture containing 16% O2 and 5.20% CO2, with N2 as the balance gas.

The protocol started with 1 min of standing while breathing through the respiratory mask, followed by 2 min of walking at 3 km/h. Treadmill speed then increased by 0.5 km/h every 30 s. Running started at approximately 8 km/h, depending on the individual transition from walking to running, and continued until volitional exhaustion or the last completed 30-s interval. Treadmill incline remained constant at 1%. Data were filtered into 30-s intervals, and the highest value in the final relevant interval was used as the maximal value. The ventilatory threshold (VT) was identified from the gas-exchange response using the V-slope method, based on the breakpoint in the relationship between carbon dioxide output (VCO₂) and oxygen uptake (VO₂) (16). After the test, participants walked at 5 km/h for 5 min while spiroergometric variables continued to be monitored.

The functional variables used in the analyses were maximal treadmill speed (Vmax), treadmill speed at the ventilatory threshold (speed at VT), maximal heart rate (HRmax), heart rate at the ventilatory threshold (HR at VT), absolute maximal oxygen uptake (VO₂max), relative maximal oxygen uptake (relative VO₂max), and relative oxygen uptake at the ventilatory threshold (VO₂ at VT). Heart rate and oxygen uptake at VT were also expressed relative to the corresponding maximal values (%HRmax and %VO₂max).

### 2.4 Refereeing quality

The criterion variable was season-long refereeing quality, calculated as the average of all final official refereeing grades received across the competitive season. During each match, the supervisor records situations involving the referee, with particular attention to actions that require a decision. Each match starts from a grade of 8.4. Correct or incorrect decisions can increase or decrease the final grade, usually by 0.1–0.5 points per decision. The present study used the grade of the main referee. Commonly evaluated events include disciplinary sanctions, penalty decisions, goals, offside situations and other match incidents that require referee intervention.

Match observers were appointed by the referee commissioner responsible for the league. Each observer evaluated several referees during the competitive season. After each round, seven members of the Croatian Football Federation Referees Committee independently reviewed the match video. The committee then discussed each match and reached a joint decision on the final grade. The final committee grade was used to calculate the refereeing quality score for each referee. This procedure reduced the influence of the initial match observer on the final grade. Individual ratings of the committee members before the final decision were not available for analysis, so inter-rater reliability could not be calculated. No formal classification of match difficulty or match importance was available for the analysed season. Match-context variables such as derby matches, team ranking or competition importance were not included in the analysis.

### 2.5 Statistical analysis

Statistical analyses were performed using STATISTICA 14.3. Arithmetic mean, standard deviation, minimum and maximum values, range, skewness and kurtosis were calculated for the study variables. Distribution normality was assessed using the Shapiro–Wilk test. Simple linear regression analyses were used to examine the association between each functional ability variable and refereeing quality. R^2^ was reported as a measure of explained variance. Spearman rank correlations were calculated because of the small sample and the non-normal distribution of refereeing quality. Spearman correlation coefficients were used for the correlation matrix. 95% confidence intervals for Spearman correlation coefficients were estimated using percentile bootstrap resampling with 100,000 resamples. To assess the influence of the lowest refereeing quality value (7.30), Spearman correlations and simple linear regressions were repeated after excluding this observation. Statistical significance was set at *p* < 0.05. A sensitivity power analysis for a two-tailed correlation was performed for the analytical sample of 16 referees. With *α* = 0.05 and statistical power of 0.80, the minimum detectable correlation was approximately |r| = 0.63.

## 3 Results

### 3.1 Functional abilities

Descriptive results for functional abilities are presented in Table 1. Mean maximal treadmill speed was 18.50 ± 1.03 km/h and mean relative VO₂max was 51.71 ± 4.28 mL/kg/min. Heart rate at VT corresponded to 91.54 ± 1.88% of HRmax, while VO₂ at VT corresponded to 87.80 ± 2.94% of VO₂max.

**Table 1:** Descriptive statistics for functional ability variables in elite football referees (*N* = 16).

Variable	Mean	SD	Min	Max	Skewness	Kurtosis
Vmax (km/h)	18.50	1.03	16.50	20.00	−0.41	−0.34
Speed at VT (km/h)	17.81	1.08	16.00	19.50	0.10	−0.90
HRmax (beats/min)	184.19	10.88	159.00	199.00	−1.35	1.74
HR at VT (beats/min)	168.63	10.92	146.00	182.00	−0.89	0.30
VO₂max (L/min)	4.38	0.36	3.76	5.28	0.74	1.40
Relative VO₂max (mL/kg/min)	51.71	4.28	42.60	58.05	−0.90	0.34
VO₂ at VT (mL/kg/min)	45.38	3.84	38.10	49.88	−0.96	−0.28
HR at VT (%HRmax)	91.54	1.88	87.83	94.65	−0.20	−0.16
VO₂ at VT (%VO₂max)	87.80	2.94	81.29	91.47	−1.10	0.48

Vmax, maximal treadmill speed; VT, ventilatory threshold; HRmax, maximal heart rate; HR at VT, heart rate at the ventilatory threshold; VO₂max, maximal oxygen uptake; VO₂ at VT, oxygen uptake at the ventilatory threshold; SD, standard deviation.

### 3.2 Refereeing quality

Descriptive statistics for refereeing quality are presented in Table 2. Mean refereeing quality was 8.21 ± 0.26 points, with values ranging from 7.30 to 8.40. Refereeing quality showed a non-normal distribution (Shapiro–Wilk W = 0.61, *p* < 0.001).

**Table 2:** Descriptive statistics for the official season-long refereeing quality grade (*N* = 16).

Variable	Mean	SD	Min	Max	Skewness	Kurtosis
Refereeing quality grade	8.21	0.26	7.30	8.40	-3.16	11.21

SD, standard deviation. The refereeing quality grade represents the mean of final official match grades obtained across the competitive season after review by the Croatian Football Federation Referees Committee.

### 3.3 Functional abilities and refereeing quality

Spearman rank correlations showed no statistically significant associations between functional ability variables and refereeing quality in the full analytical sample (Table 3).

**Table 3:** Spearman rank correlation matrix for functional ability variables and refereeing quality (*N* = 16).

Variable	1	2	3	4	5	6	7	8
1. Refereeing quality	1.000							
2. Vmax	−0.181	1.000						
3. Speed at VT	−0.372	0.756***	1.000					
4. HRmax	0.090	−0.391	−0.123	1.000				
5. HR at VT	0.228	−0.396	−0.149	0.902***	1.000			
6. VO₂max	0.386	0.420	0.061	0.004	−0.144	1.000		
7. Relative VO₂max	−0.168	0.530*	0.245	−0.382	−0.449	0.545*	1.000	
8. VO₂ at VT	0.118	0.447	0.119	−0.419	−0.384	0.459	0.829***	1.000

Values are Spearman's ρ. Vmax, maximal treadmill speed; VT, ventilatory threshold; HRmax, maximal heart rate; VO₂max, maximal oxygen uptake. * *p* < 0.05; ** *p* < 0.01; *** *p* < 0.001. For correlations with refereeing quality (column 1), 95% percentile bootstrap confidence intervals were: Vmax −0.619 to 0.341; speed at VT −0.800 to 0.187; HRmax −0.485 to 0.626; HR at VT −0.349 to 0.711; VO₂max −0.139 to 0.756; relative VO₂max −0.669 to 0.369; VO₂ at VT −0.494 to 0.653. Confidence intervals were based on 100,000 bootstrap resamples.

Correlation coefficients ranged from -0.372 to 0.386 (all *p* ≥ 0.140), and all 95% bootstrap confidence intervals included zero. Simple regression analyses showed the same pattern, with no statistically significant predictors of refereeing quality and low explained variance (R^2^ = 0.001–0.090; Table 4). After exclusion of the lowest refereeing quality value (7.30; *n* = 15), six of the seven Spearman associations remained non-significant (all *p* ≥ 0.229). Absolute VO₂max showed a statistically significant positive correlation with refereeing quality (*ρ* = 0.532, 95% CI 0.054 to 0.822, *p* = 0.041), while the corresponding linear regression remained non-significant (R^2^ = 0.157, *p* = 0.143).

**Table 4:** Simple regression analyses of functional ability variables and refereeing quality (*N* = 16).

Variable	B	95% CI	SE	R^2^	t	p
Vmax (km/h)	−0.028	−0.171 to 0.116	0.067	0.012	−0.410	0.688
Speed at VT (km/h)	−0.072	−0.205 to 0.060	0.062	0.090	−1.174	0.260
HRmax (beats/min)	−0.002	−0.016 to 0.011	0.006	0.009	−0.349	0.732
HR at VT (beats/min)	0.002	−0.011 to 0.016	0.006	0.010	0.384	0.707
VO₂max (L/min)	0.016	−0.396 to 0.428	0.192	0.001	0.084	0.934
Relative VO₂max (mL/kg/min)	−0.012	−0.047 to 0.022	0.016	0.042	−0.784	0.446
VO₂ at VT (mL/kg/min)	−0.003	−0.042 to 0.036	0.018	0.002	−0.167	0.870

B, unstandardised regression coefficient; CI, confidence interval; SE, standard error; R^2^, coefficient of determination; Vmax, maximal treadmill speed; VT, ventilatory threshold; HRmax, maximal heart rate; VO₂max, maximal oxygen uptake. Statistical significance was set at *p* < 0.05.

## 4 Discussion

The main result of this study was that no statistically significant association was found between laboratory-measured functional abilities and refereeing quality in elite football referees. The full-sample Spearman analysis showed the same overall pattern. The study hypothesis was not supported. The small analytical sample limits the detection of weak and moderate associations. Smaller relationships between functional capacity and refereeing quality cannot be excluded.

A similar result has previously been reported in top-level handball referees. Belcic et al. found no statistically significant relationship between functional abilities and refereeing quality in 31 elite handball referees and linked the result to the homogeneity created by selection and elimination criteria (17). The present football data follow the same pattern. Referees who reach the highest national level have already passed several years of selection, physical testing and performance evaluation. Physical fitness may act mainly as a prerequisite for reaching and remaining at this competition level. This interpretation should be considered together with the small sample, restricted variability in refereeing grades and limited statistical power.

The functional results confirm that the present group was well prepared. Mean relative VO₂max was 51.71 mL/kg/min, almost identical to the value of 51.9 mL/kg/min reported for elite male football referees in the systematic review by Martinho et al. (3). Absolute VO₂max in the present study was 4.38 L/min, compared with approximately 3.98 L/min reported for elite male referees in the same review. Mean Vmax was 18.50 km/h and mean HRmax was 184.19 beats/min. These values support the interpretation that the participants already had aerobic characteristics expected from referees working at a high competition level. Laboratory testing was performed in January, between the first and second parts of the competitive season, while refereeing quality was calculated from official grades across the competitive season. The analysed referees had passed the required physical fitness tests before the second part of the season and followed prescribed training programmes during the competition period. Functional abilities were assessed only once. Possible changes in physical condition across the season were not measured.

High functional capacity is still needed because match demands are substantial. Recent high-level data show that main referees cover approximately 9.4–11.6 km and perform repeated high-intensity actions, while heart rate can reach very high relative values during specific periods of match play (4). Elite referee data also show clear peak running and physiological demands that can be missed when only match averages are considered (6). Professional Spanish referees are exposed to repeated high-speed actions, accelerations and decelerations throughout matches (5). A laboratory test may describe the referee's available physiological capacity without directly representing the short periods in which the most difficult decisions are made.

The present result differs partly from Castillo-Rodríguez et al. (12). In their study of 22 football referees, physical fitness was related to total distance and to the percentage of successful decisions, and the overall physical fitness score predicted decision-making performance. The outcome used in the present study was different. Refereeing quality was based on official grades accumulated across a competitive season and reviewed by an expert committee, while Castillo-Rodríguez et al. analysed the success of individual decisions. The range of quality grades in the present sample was only 7.30–8.40. A restricted criterion range makes it more difficult for a physiological variable to show a statistical relationship even when physical preparation is practically important.

The findings of McEwan et al. give another explanation for the lack of a statistically significant association with general laboratory fitness (18). Decision errors were more frequent when referees had a heart rate of at least 90% of HRmax, a respiratory rate of at least 80% of peak respiratory rate, or very high running speeds immediately before a decision. Their results suggest that acute load in the seconds preceding a decision can be more relevant to decision accuracy than a general measure of aerobic capacity recorded on a separate day. Vigorous aerobic exercise can also acutely change some basic perceptual-cognitive measures without necessarily changing higher-order refereeing decision performance (19). A referee can have a high VO₂max and still experience short match periods in which physiological strain affects the quality of information processing.

Refereeing quality also depends on cognitive and psychological characteristics. Expert and novice referees differ in decision-making strategy, accuracy and confidence during disciplinary decisions (13). Anxiety and pressure can change the cognitive processing used during football decisions (14). Response-suppression ability has recently been associated with more accurate disciplinary decision-making, even when overall decision accuracy did not clearly differ between referee levels (20). Mental fatigue increases perceived effort during a football-referee match simulation, but did not necessarily reduce inhibitory control in the tested task (21). Variability in judgment is also present when referees evaluate comparable football situations, which adds another source of variation to season-long performance grades (22). These findings support the view that final refereeing quality is produced by several interacting components and cannot be reduced to one physiological characteristic.

Match context changes the physical work required from the referee. Martínez-Torremocha et al. showed that elite referees adapt their running performance to the physical, technical and tactical characteristics of the match, with experience contributing to this adaptation (23). Main and assistant referees also show different external and internal loads (7). A referee with strong laboratory results may not use that capacity in the same way in every match because game pace, tactical structure, transitions and positioning demands change from one competition to another. This is one reason why field performance and laboratory capacity should be interpreted together rather than as interchangeable measures. Refereeing experience may influence positioning, decision-making and adaptation to match demands. The present sample had a median of 6 years of experience in the highest Croatian league. The small sample limited the inclusion of several potential confounding variables in the regression models. The field-based outcome in the present study was the official refereeing grade. Direct match-based measures of physical performance, such as GPS-derived running load, heart rate, accelerations and decelerations, were not included. Laboratory capacity and match-specific physical performance should not be interpreted as equivalent measures.

Training should continue to develop the physiological capacity needed for repeated high-intensity match actions. Repeated-sprint training can improve high-intensity physical performance (24), and the intermittent structure of refereeing supports programmed high-intensity work alongside aerobic conditioning. Physical training alone is not enough for the complete refereeing task. Referees should also practise decision-making while physically loaded and use football-specific perceptual-cognitive training. A recent systematic review and meta-analysis found a large positive effect of video-based training on football referees’ decision-making skills (25). Combining physical and decision training is a practical way to prepare referees for the periods when high running demands and important decisions occur at the same time.

The study has a clear strength in the level and completeness of the initial sample. All 17 referees eligible to officiate in the highest Croatian league during the 2022/2023 season entered the testing procedures, while 16 completed the physiological assessment. Ten referees were listed internationally by UEFA and/or FIFA. The official quality measure was based on an entire season and was reviewed by seven experts rather than relying on one isolated laboratory decision task. The same characteristics also create limitations. The complete referee list from the highest Croatian national competition was included, but the absolute analytical sample was small. The sensitivity analysis showed that the study had sufficient power mainly for large associations. Weak and moderate associations may have remained undetected. Refereeing quality had a restricted and markedly non-normal distribution. The grading system may create a ceiling effect around the standard starting grade of 8.4, as the highest season-long mean in the present sample was 8.40. This grading structure may compress between-referee variability at the upper end of the scale. The sensitivity analysis excluding the lowest refereeing grade produced a statistically significant Spearman correlation only for absolute VO₂max, while the corresponding linear regression remained non-significant. This indicates that this association was sensitive to a single observation and should be interpreted cautiously. Individual ratings of the seven committee members were not available, so inter-rater reliability could not be calculated. No formal classification of match difficulty or importance was available and match-context variables were not included in the analysis. The exact number of matches contributing to the final season grade for each referee was not available in the archived dataset. Differences in the number of evaluated matches may have affected the precision of the mean refereeing grade. Functional abilities were assessed once in January. Possible changes in functional capacity during the competitive season were not measured. Direct match-based physiological and running-load measures were not included. A broader testing battery could include repeated-sprint ability, short sprint performance, acceleration and deceleration capacity, change-of-direction ability and the capacity to maintain high-intensity performance. High-speed activity in elite referees changes during match play and may not be adequately represented by general aerobic measures alone (26).

Future studies should include repeated physiological assessments across the competitive season and direct match-based measures of physical load. GPS-derived running performance, heart rate, sprint ability, repeated high-intensity efforts, acceleration, deceleration and change-of-direction ability could provide a more complete description of referee physical performance. Larger samples would allow adjustment for refereeing experience, match context and other potential confounding variables.

## 5 Conclusion

No statistically significant association was found between the examined functional ability variables and refereeing quality in the full sample of elite football referees. The full-sample Spearman analysis showed the same overall pattern. Exclusion of the lowest refereeing grade changed the Spearman result for absolute VO₂max, indicating sensitivity to individual observations. The small sample limits the detection and stability of weak and moderate associations.

High physical fitness remains important for meeting elite match demands. Laboratory-measured functional capacity alone did not show a clear association with refereeing quality in the present sample. Larger samples, repeated physiological testing and direct match-based physical measures are needed to examine this relationship more precisely.

Physical preparation remains an important component of high-level refereeing. Training programmes should maintain high aerobic and repeated high-intensity capacity while also exposing referees to decision-making under match-like physical load.

## Data Availability

The raw data supporting the conclusions of this article will be made available by the authors, without undue reservation.

## References

[1] Stølen T Chamari K Castagna C Wisløff U. Physiology of soccer. Sports Med. (2005) 35:501–36. 10.2165/00007256-200535060-00004 15974635

[2] Bangsbo J Mohr M Krustrup P. Physical and metabolic demands of training and match-play in the elite football player. J Sports Sci. (2006) 24:665–74. 10.1080/02640410500482529 16766496

[3] Martinho DV Field A Rebelo A Gouveia ER Sarmento H. A systematic review of the physical, physiological, nutritional and anthropometric profiles of soccer referees. Sports Med Open. (2023) 9:72. 10.1186/s40798-023-00610-7 37561241 PMC10415246

[4] Zhang L Geok SK Wazir MRWN Qin L. Physical demands and physiological response of soccer referees in high-level matches: a systematic review. PLoS One. (2025) 20:e0315403. 10.1371/journal.pone.0315403 39854410 PMC11759383

[5] Martínez-Torremocha G Martin-Sanchez ML Garcia-Unanue J Felipe JL Moreno-Pérez V Paredes-Hernández V. Physical demands on professional Spanish football referees during matches. Sci Med Footb. (2023) 7:139–45. 10.1080/24733938.2022.2064539 35392769

[6] Yousefian F Zafar A Peres P Brito J Travassos B Figueiredo P. Intensity demands and peak performance of elite soccer referees during match play. J Sci Med Sport. (2023) 26:58–62. 10.1016/j.jsams.2022.10.006 36344363

[7] Gomes R Mendes R Ferreira A Mendes R Dias G Martins F. Physical and physiological demands of amateur Portuguese field and assistant football referees. Sports. (2024) 12:133. 10.3390/sports12050133 38787002 PMC11126070

[8] Mallo J Frutos PG Juárez D Navarro E. Effect of positioning on the accuracy of decision making of association football top-class referees and assistant referees during competitive matches. J Sports Sci. (2012) 30:1437–45. 10.1080/02640414.2012.711485 22867266

[9] Vater C Schnyder U Müller D. That was a foul! how viewing angles, viewing distances, and visualization methods influence football referees’ decision-making. Ger J Exerc Sport Res. (2024) 54:476–85. 10.1007/s12662-024-00947-5

[10] Castagna C Abt G D’Ottavio S. Physiological aspects of soccer refereeing performance and training. Sports Med. (2007) 37:625–46. 10.2165/00007256-200737070-00006 17595157

[11] Krustrup P Bangsbo J. Physiological demands of top-class soccer refereeing in relation to physical capacity: effect of intense intermittent exercise training. J Sports Sci. (2001) 19:881–91. 10.1080/026404101753113831 11695510

[12] Castillo-Rodríguez A Alejo-Moya EJ Figueiredo A Onetti-Onetti W González-Fernández FT. Influence of physical fitness on decision-making of soccer referees throughout the match. Heliyon. (2023) 9:e19702. 10.1016/j.heliyon.2023.e19702 PMC10558927 37809542

[13] Wang H Zhang C Ji Z Li X Wang L. Faster, more accurate, more confident? An exploratory experiment on soccer referees’ yellow card decision-making. Front Psychol. (2024) 15:1415170. 10.3389/fpsyg.2024.1415170 39144600 PMC11322511

[14] Zhang L Shi H Zhang H Ding J Wang Z. How do anxiety and stress affect soccer referees? An ERPs study. Front Psychol. (2024) 15:1294864. 10.3389/fpsyg.2024.1294864 38993335 PMC11236741

[15] Vucetic V. Dijagnostički postupci za procjenu energetskih kapaciteta sportasa. In: Jukic I Milanovic D Salaj S Gregov C, editors. Kondicijska Priprema Sportasa 2009: Trening Izdrzljivosti. Zagreb: Faculty of Kinesiology, University of Zagreb; Udruga kondicijskih trenera Hrvatske (2009). p. 20–31.

[16] Beaver WL Wasserman K Whipp BJ. A new method for detecting anaerobic threshold by gas exchange. J Appl Physiol. (1986) 60:2020–7. 10.1152/jappl.1986.60.6.2020 3087938

[17] Belcic I Ruzic L Marosevic A. Influence of functional abilities on the quality of refereeing in handball. Balt J Health Phys Act. (2020) 12:23–34. 10.29359/BJHPA.12.3.03

[18] McEwan GP Unnithan VB Easton C Glover AJ Arthur R. Decision-making accuracy of soccer referees in relation to markers of internal and external load. Eur J Sport Sci. (2024) 24:659–69. 10.1002/ejsc.12096 38874944 PMC11235990

[19] Albaladejo-García C Caballero C Asencio P Moreno FJ. Acute effects of a vigorous-intensity warm-up on response suppression and decision-making of football referees. J Sports Sci. (2024) 42:1081–9. 10.1080/02640414.2024.2383071 39077984

[20] Albaladejo-García C Ignoul E Demarez M Caballero C Helsen WF Moreno FJ. Response suppression and decision-making in association football referees. Psychol Sport Exerc. (2025) 80:102908. 10.1016/j.psychsport.2025.102908 40490237

[21] Lopes de Lima CZ De Sousa Fortes L Pires DA Souza Machado HE Socorro Dantas Oliveira AD Coswig VS. Mental fatigue increases perception of effort but does not compromise the inhibitory control of football referees during a match simulation physical task. Eur J Sport Sci. (2025) 25:e12222. 10.1002/ejsc.12222 39817859 PMC11829703

[22] Samuel RD Galily Y Hochman G. The ’silent’ noise: moving forward from bias to noise in football referees’ decision-making. Int J Sport Exerc Psychol. (2026) 24:405–30. 10.1080/1612197X.2025.2453462

[23] Martínez-Torremocha G Alonso-Callejo A Garcia-Unanue J Gallardo L Sanchez-Sanchez J Felipe JL. How do technical and tactical demands of football matches affect the physical performance of elite football referees? Exploring the role of experience. J Strength Cond Res. (2024) 38(11):e664–9. 10.1519/JSC.0000000000004902 39454193

[24] Krakan I Milanović L Belcic I. Effects of plyometric and repeated sprint training on physical performance. Sports. (2020) 8:91. 10.3390/sports8070091 32605121 PMC7404803

[25] Zhou R Hao X Deng P Li D Song Y Zhang Y. The impact of video-based training on football referees’ decision-making skills: a systematic review and meta-analysis. BMC Sports Sci Med Rehabil. (2025) 17:186. 10.1186/s13102-024-01046-6 40629454 PMC12239248

[26] Amatori S Helsen WF Baldari C Serra T Belli A Guidetti L. High-speed efforts of elite association football referees in national and international matches. J Strength Cond Res. (2024) 38(8):e417–22. 10.1519/JSC.0000000000004821 39072662

